# The Wound Healing Effect of Doxycycline after Corneal Alkali Burn in Rats

**DOI:** 10.1155/2019/5168652

**Published:** 2019-10-10

**Authors:** Qiong Yi, Wen-jin Zou

**Affiliations:** Department of Ophthalmology, First Affiliated Hospital of Guangxi Medical University, Nanning, Guangxi Zhuang Autonomous Region 530021, China

## Abstract

**Purpose:**

To evaluate the wound healing effect of doxycycline and its underlying mechanisms in a rat model of corneal alkali burn.

**Methods:**

Male SD rats were administered 1.0 N NaOH in the right cornea for 25 seconds and randomly divided into the doxycycline group and the control group, with 84 rats in each group. 1.0 g·L^−1^ doxycycline eye drops (doxycycline group) or vehicle (control group) was topically instilled onto the rat cornea after chemical injury. Three days, 7 days, and 14 days after alkali burn, microscopy was used to observe corneal wound healing by fluorescein staining and the degree of corneal opacity. The expression of transforming growth factor-beta 1 (TGF-*β*1) and matrix metalloproteinase-9 (MMP-9) was detected by RT-PCR and ELISA, alpha-smooth muscle actin (a-SMA) levels were measured by immunofluorescent staining, and Western blot assays for TGF-*β*1, a-SMA, and nuclear factor-kappa B (NF-*κ*B) were also performed.

**Results:**

Corneal wound healing and corneal opacity scores were better in the doxycycline group than in the control group. Three, 7, and 14 days after corneal alkali burn, a significant increase in TGF-*β*1 was observed in corneas from the control group, compared with the corneas from the doxycycline group (*P* < 0.05). The corneal levels of MMP-9 in the doxycycline group were lower than those in the control group 3 days and 7 days after alkali burn (*P* < 0.05). In addition, doxycycline inhibited *α*-SMA expression and suppressed NF-*κ*B expression.

**Conclusion:**

Doxycycline treatment promoted corneal healing and reduced corneal opacity in SD rats. Doxycycline protected the cornea from alkali burn injury by reducing TGF-*β*1, MMP-9, NF-*κ*B, and *α*-SMA expression.

## 1. Introduction

Chemical injury of the eye is a common intractable ocular disease in the clinic. Slow epithelialization, persistent ulceration, corneal perforation, neovascularization (NV), and opacification are the main complications, and chemical injury of the eye may cause permanent visual impairment or blindness [1, 2]. Keratocyte activation, myofibroblast formation, and tissue fibrosis all participate in the wound healing process or scar formation in the cornea after alkali burn [3, 4]. Therefore, corneal scars may be reduced by inhibiting all or one of the above three processes.

Normal corneal keratocytes are stationary and help maintain corneal transparency; nevertheless, after corneal injury, the disruption of corneal integrity induces quiescent keratocytes to differentiate into active myofibroblasts [5]. The morphology of corneal myofibroblasts is different than that of keratocytes, and corneal myofibroblasts are defined by their unique expression of alpha-smooth muscle actin (*α*-SMA) [6]. Among the many cytokines and growth factors that affect the corneal wound healing reaction, transforming growth factor-*β*1 (TGF-*β*1) plays an important role in the regeneration of damaged tissue [7, 8]. After corneal alkali burn, TGF-*β*1 has been shown to be upregulated and induce the differentiation of keratocytes to myofibroblasts, which contributes to the recovery of the cornea [9, 10]. TGF-*β*1 has also been reported as an effective chemotactic factor for monocytes, neutrophils, and macrophages [11, 12]. During the inflammatory response, TGF-*β*1 has been found to stimulate the expression of MMP-9 in the cornea [13]. In addition, NF-*κ*B competes with TGF-*β*1 to control the expression of MMPs [14], and the degradation of the basement membrane by MMP-9 is known to contribute to the promotion of pathogenic ulcers and the perforation of the corneal stroma [15]. NF-*κ*B is mediated angiogenesis, which affects the transparency of the cornea [16]. The overexpression of TGF-*β*1 has been shown to induce the accumulation of myofibroblasts, and this is believed to be a major mechanism of corneal opacification after damage, as myofibroblasts exhibit a different crystalline production and are, consequently, opaquer than keratocytes [17].

Doxycycline (Doxy) was discovered in the early 1960s as a long-acting, low-cost, semisynthetic tetracycline antibiotic that has a broad-spectrum antibacterial effect [18]. Doxycycline has attracted much attention due to its used in the treatment of various diseases such as pulmonary fibrosis, nasal polyps, and chronic periodontitis [19–21]. In addition, doxycycline has been reported to inhibit the remodeling of skin fibroblasts and human conjunctival fibroblasts [22, 23]. In treating corneal diseases, tetracyclines inhibit the collagenolytic degradation of the cornea resulting from chemical injury and other noninfectious corneal ulcers by inhibiting MMP activity [24]. It has been reported that doxycycline may reduce the formation of corneal scars by inhibiting the infiltration of inflammatory cells and neovascularization after corneal alkali burn in rats [25, 26]. However, the effect of doxycycline in corneal scarring and the underlying molecular mechanism are still unclear. In this study, we aimed to evaluate the effect of doxycycline on corneal wound healing and the associated mechanism after alkali burn.

## 2. Materials and Methods

### 2.1. Animals

All animals were treated according to the Association for Research in Vision and Ophthalmology (ARVO) Statement for the Use of Animals in Ophthalmic and Vision Research, and the procedures were approved by the Guangxi Medical University of Medicine Institutional Animal Care and Use Committee. Eighty-four male Sprague-Dawley (SD) rats (body weight range, 180–220 g) were divided into two groups, with 42 rats in each group.

### 2.2. In Vivo Animal Model of Corneal Chemical Burn Injury

Before the establishment of the alkali burn model, an antibiotic (0.3% tobramycin eye drops) was administered 4 times a day to prevent corneal infection, and the presence of disease of the anterior segment was checked with a slit lamp before the experiment. The rats were anaesthetized by an intraperitoneal injection of ketamine (40 mg/kg)/xylazine (8 mg/kg). Using tetracaine hydrochloride eye drops as a topical anesthetic, cotton was used to swab the conjunctival sac fluid. A home-made alkali burn mold was made according to the following procedure: the tip of a 200 *µ*L pipette tip was cut horizontally 15 mm from the end; the reminder of the pipette tip was filled with enough cotton, so that cotton was exposed at the tip of the pipette tip and formed a flat and slightly convex surface with moderate tightness; the tip was sterilized by high temperatures before use; 20 *µ*L of 1.0 N NaOH was added to the mold (circular, 3.0 mm diameter); and the mold was placed vertically in the center of the cornea of the right eye of each SD rats for 25 seconds to produce a corneal burn. The wound surface was rapidly washed with 20 mL 0.9% physiological saline for 1 min. Doxycycline eye drops were made by dissolving doxycycline powder (Sigma-Aldrich, St. Louis, MO, USA) in PBS. The doxycycline group was treated with 1.0 g·L^−1^ doxycycline eye drops four times a day for 14 days, and the control group was treated with PBS in the same manner. Alkali burn occurred on day 0, and the animals were euthanized 3, 7, and 14 days after injury.

### 2.3. Corneal Opacity and Injury

The degree of corneal opacity was examined using a microscope on days 3, 7, and 14 after corneal alkali burn. The degree of haze was scored clinically on the following 0–4 numerical scale: 0, clear cornea; 1, slight stromal opacity; 2, moderate stromal opacity; 3, severe corneal opacity with difficultly viewing the iris; and 4, opaque cornea without the ability to view the iris. Epithelial defect was assessed 3, 7, and 14 days after chemical injury by staining with 1% fluorescein and imaging the staining. Two independent masked observers analyzed the images. The area of the epithelial defect was calculated by Image-Pro Plus® software.

### 2.4. Real-Time PCR

Eight rats were randomly selected from each group on days 3, 7, and 14 after chemical injury, and the corneal tissue was aseptically removed along the corneal scleral margin. The corneal tissue was divided into two equal parts, and one portion was quickly stored in a −80°C freezer, and the other was homogenized in a 1.5 ml centrifuge tube containing cracking Buffer RL. Then, Trizol reagent (TaKaRa, Tokyo, Japan) was used to collect total RNA. The RNA was reverse-transcribed into cDNA using a PrimeScript RT Master Mix reverse transcription kit (TaKaRa, Tokyo, Japan). Real-time polymerase chain reaction (RT-PCR) was performed using Premix Ex Taq gene expression assays (TaKaRa, Tokyo, Japan). The following primers were used: GAPDH forward, TGAAGGTCGGTGTGAACGGATTTG; GAPDH reverse, GTTGAATTTGCCGTGAGTGGAGTC; TGF-*β*1 forward, CATTGCTGTCCCGTGCAGA; TGF-*β*1 reverse, AGGTAACGCCAGGAATTGTTGCTA; MMP-9 forward, AGCCGGGAACGTATCT GGA; and MMP-9 reverse, TGGAAACTCACACGCCAGAA G. CT values were used for the relative quantification of the initial template. The final results are expressed as 2^−ΔΔct^.

### 2.5. Enzyme-Linked Immunosorbent Assay (ELISA)

The other pieces of corneal tissue collected on days 3, 7, and 14 were removed from the −80°C freezer, homogenized in PBS, stored at −20°C overnight, frozen and thawed twice, and then centrifuged at 500 ×*g* 4°C for 5 min in a low-temperature centrifuge. TGF-*β*1 and MMP-9 levels in the supernatant were quantified by ELISA according to the instructions of the kit (Cusabio Biotech, Wuhan, China). The optical absorbance (OD) value of each well was measured at a wavelength of 450 nm with microplate reader. A standard curve was drawn using professional software (Curve Expert 1.4), and the OD values were used to determine the corresponding protein concentration from the standard curve.

### 2.6. Immunofluorescence Assay

Paraffin sections (5 *µ*m) were processed for immunofluorescent staining for *α*-SMA. After high-pressure repair, the sections were blocked with 5% goat serum for 30 min. Then, the sections were incubated for 1 hr with a rabbit polyclonal antibody against *α*-smooth muscle actin (ab5694, 1 : 200; Abcam, Cambridge, MA, USA) at room temperature. A FITC-conjugated antibody against goat immunoglobulin (DyLight 649, 1 : 500; Fudebio-tech, Hangzhou, China) was added to the specimens at room temperature for 1 hr, An antifluorescence quenching sealant containing DAPI (Solarbio, Beijing, China) was used to seal and counterstaining the sections, and photomicrographs were captured by Olympus fluorescence microscopy.

### 2.7. Western Blotting for *α*-SMA, TGF-*β*1, and NF-*κ*B

The corneas treated with 1.0 g·L^−1^ doxycycline eye drops or PBS for 3, 7, and 14 days were excised and homogenized in RIPA lysis buffer (Beyotime, Shanghai, China) containing proteinase inhibitors using an ultrasound homogenizer. Three corneas were used for each experimental condition. The samples were diluted with one part 4× sample buffer (Beyotime, Shanghai, China) and boiled for 5 min. The same amount of total protein from each cornea was separated by 8% SDS-PAGE and then transferred to PVDF membranes (0.45 *µ*m, Millipore, Bedford, MA, USA), followed by incubation overnight with primary antibodies against *α*-SMA (ab5694, 1 : 100; Abcam, Cambridge, MA, USA), TGF-*β*1 (ab92486, 1 : 200; Abcam, Cambridge, MA, USA), NF-*κ*B (D14E12, 1 : 500; Cell Signaling Technology, Danvers, MA, USA), and GAPDH (FD0063, 1 : 1000; FuDe Biological Technology, Hangzhou, China). Subsequently, the membranes were washed in TBS and then with an anti-rabbit secondary antibody (ab6721, 1 : 2000; Abcam, Cambridge, MA, USA) for 1 h at room temperature. Finally, the membranes were developed and photographed.

### 2.8. Statistical Analysis

The data are expressed as the mean ± standard deviation (SD). Student's *t*-test was applied to identify the differences between the groups, and *P* values <0.05 were considered statistically significant.

## 3. Results

### 3.1. Degree of Corneal Opacity and Injury

**Figure 1:**
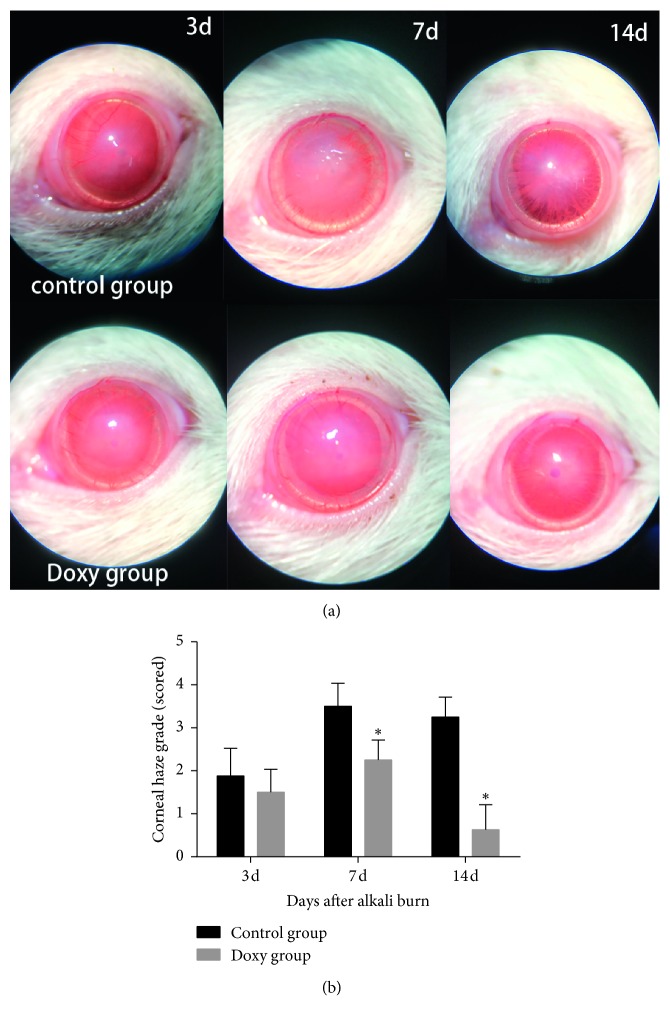
(a) Slit-lamp photographs of corneas in the doxycycline group and the control group on days 3, 7, and 14 after corneal alkali burn. (b) Corneal opacity scores of two groups on the 3rd, 7th, and 14th day. The doxycycline group had a significant lower score than the control group 7 and 14 days after chemical injury (*P* < 0.05). ^*∗*^Statistically significant; data are expressed as mean ± SD, *n* = 8.

**Figure 2:**
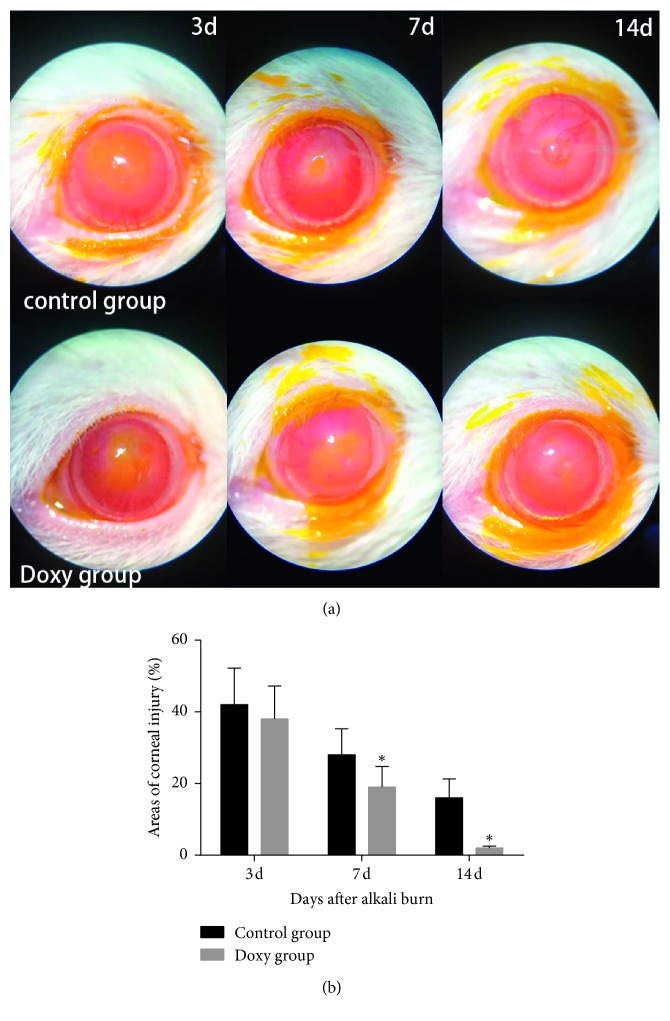
(a) Slit-lamp photographs of fluorescein-stained corneas in the doxycycline group and the control group 3, 7, and 14 days after corneal alkali burn. (b) The corneal epithelial defect areas percentage of two groups on days 3, 7, and 14 after corneal alkali burn. There was a significant difference on days 7 and 14 after corneal alkali burn between two groups (*P* < 0.05). ^*∗*^Statistically significant; data are expressed as mean ± SD, *n* = 8.

Representative pictures of the injured corneas are shown in Figure 1(a). The corneal opacity score was lower in the doxycycline group than in the control group 7 and 14 days after chemical injury (*P* < 0.05) (Figure 1(b)). In addition, after 7 and 14 days, the corneal epithelial defects in the doxycycline group were also less severe than those in the control group (*P* < 0.05) (Figure 2).

### 3.2. Real-Time PCR

**Figure 3:**
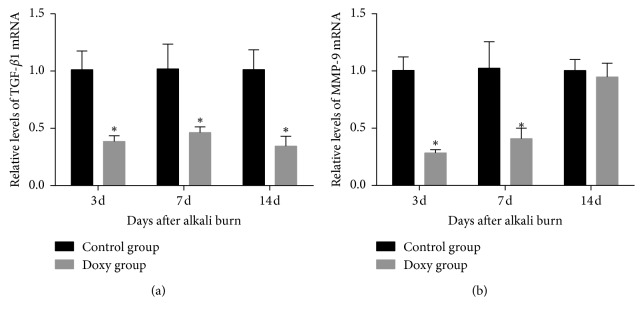
Real-time PCR of the relative expression of transforming growth factor-*β*1 (TGF-*β*1) (a) and matrix metalloproteinase-9 (MMP-9) (b) mRNA in the doxycycline and control group 3, 7, and 14 days after corneal alkali burn. Compared with the control group, TGF-*β*1 and MMP-9 mRNA expression in the doxycycline group were significantly decreased 3 and 7 days after corneal alkali burn, but just TGF-*β*1 mRNA expression decreased on day 14 after injury (*P* < 0.05). ^*∗*^Statistically significant; data are expressed as mean ± SD, *n* = 8.

Real-time PCR showed that the levels of TGF-*β*1 mRNA were lower in the doxycycline group than in the control group 3, 7, and 14 days after corneal alkali burn (*P* < 0.05) (Figure 3(a)). However, MMP-9 mRNA expression was lower in the doxycycline group than in the control group 3 and 7 days after injury (*P* < 0.05) (Figure 3(b)). The difference between the two groups was no statistically significant 14 days after corneal alkali burn (*P* > 0.05).

### 3.3. ELISA

**Figure 4:**
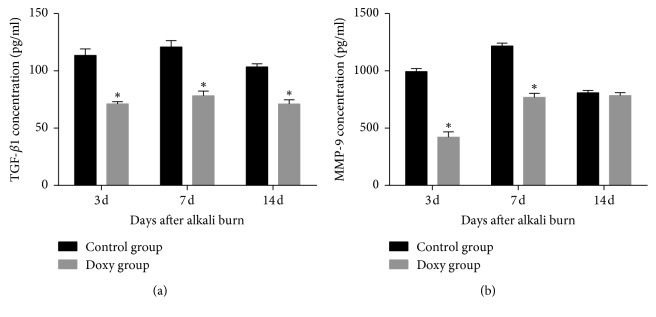
Transforming growth factor-*β*1 (TGF-*β*1) (a) and matrix metalloproteinase-9 (MMP-9) (b) concentrations in the doxycycline group and control group 3, 7, and 14 after corneal alkali burn. The doxycycline group showed a significant decrease in the TGF-*β*1 and MMP-9 concentrations compared with control group on days 3 and 7 after injury, but just TGF-*β*1 concentrations decreased on day 14 (*P* < 0.05). ^*∗*^Statistically significant; data are expressed as mean ± SD, *n* = 8.

The TGF-*β*1 levels in the doxycycline group (71.18 ± 1.88, 78.13 ± 4.23, and 71.08 ± 3.59 pg/ml) were significantly lower than those in the control group (113.38 ± 5.80, 120.80 ± 5.67, and 103.46 ± 2.75 pg/ml) 3, 7, and 14 days after alkali burn (*P* < 0.05) (Figure 4(a)). At the same time, the MMP-9 levels in the doxycycline group (420.18 ± 46.36 and 767.63 ± 35.01 pg/ml) were lower than those in the control group (993.27 ± 26.96 and 1217.42 ± 25.05 pg/ml) 3 and 7 days after injury (*P* < 0.05) (Figure 4(b)). However, there was no statistically significant difference between doxycycline group (784.70 ± 24.92 pg/ml) and control group (807.56 ± 21.22 pg/ml) 14 days after chemical injury (*P* > 0.05).

### 3.4. Immunofluorescence

**Figure 5:**
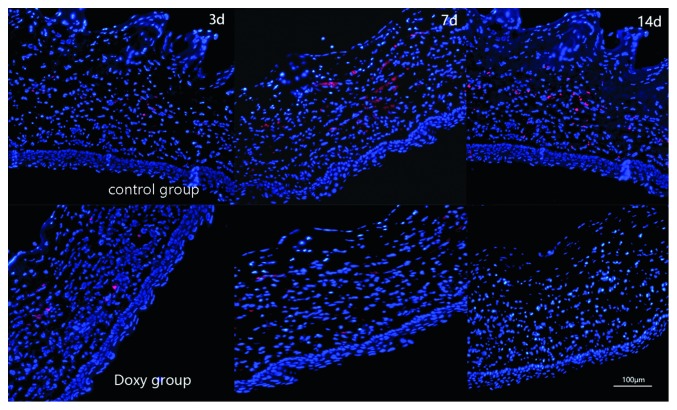
Immunofluorescence staining in the doxycycline group and the control group 3, 7, and 14 days after corneal alkali burn. DAPI was used for nuclear staining (blue). Immunofluorescent staining for *α*-SMA was weaker than in the control group 3, 7, and 14 days after injury.

The immunofluorescent staining of *α*-SMA in the doxycycline and control groups is shown in Figure 5. The doxycycline group expressed less red fluorescence than that in the control group 3, 7, and 14 days after injury. In other words, the immunofluorescent staining for *α*-SMA was weaker in the doxycycline group than in the control group. The nuclei were stained blue with DAPI dye. Doxycycline treatment reduced *α*-SMA expression in the alkali-burned corneas.

### 3.5. Western Blotting

**Figure 6:**
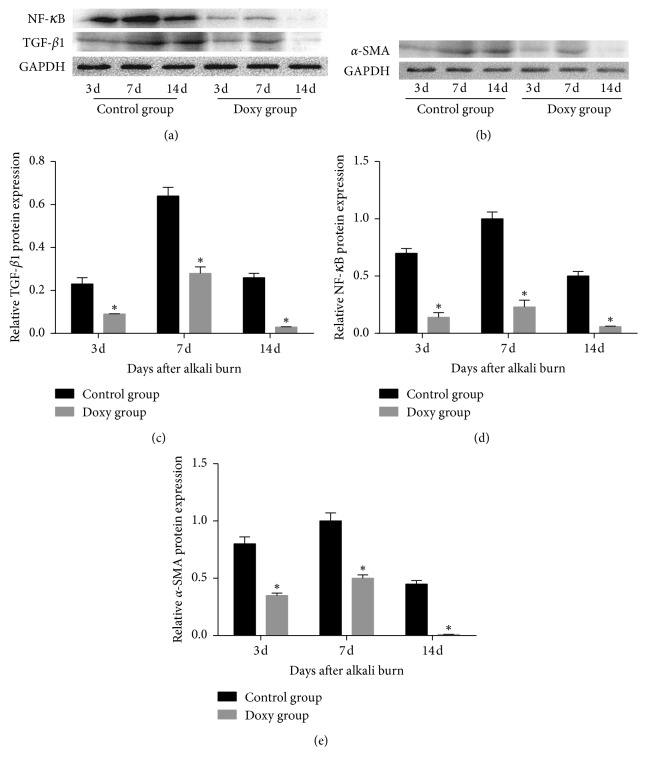
Western blot of the relative expression of transforming growth factor-*β*1 (TGF-*β*1), nuclear factor-kappa B (NF-*κ*B), and anti-*α*-smooth muscle actin (*α*-SMA) protein. The expression of these three proteins in the doxycycline group was significantly decreased compared to that in the control group 3, 7, and 14 days after chemical injury (*P* < 0.05). ^*∗*^Statistically significant; data are expressed as mean ± SD, *n* = 3.

Western blot analysis was also used to quantify the levels of TGF-*β*1, *α*-SMA, and NF-*κ*B expression. When normalized to GAPDH expression, the expression of these three proteins in the doxycycline group was significantly decreased compared to that in the control group 3, 7, and 14 days after chemical injury (Figure 6).

## 4. Discussion

The cornea is the refractive system of the eye and acts as a barrier. Chemical damage to the eye accounts for 11.5% and 22.1% of ocular injuries. Corneal scarring caused by corneal trauma and ulcers is the leading contributor to corneal blindness [27, 28]. Currently, penetration and lamellar keratoplasty are the main clinical methods for treating corneal scars. However, due to the lack of cornea donors and for economic reasons, many patients cannot receive surgical treatment and eventually lose their vision. This emphasizes the need for developing new and more effective treatment strategies.

In recent years, the application of doxycycline has been widely investigated in ophthalmology. Peng et al. [29] treated laser-injured mice with intraperitoneal injections of doxycycline, and the results indicated that doxycycline can inhibit fibrosis in choroidal neovascularization. Other studies have also shown that doxycycline enhances allograft survival in alkali-burned mouse corneas by inhibiting corneal neovascularization and inflammation in the corneal bed [30]. Wang et al. [31] treated corneal erosion syndrome patients with a combination of oral doxycycline and topical corticosteroids, and 71% of patients were symptom-free. McElvanney [32] treated pseudomonas keratitis with oral doxycycline, and the results suggested that doxycycline may help stabilize corneal rupture and prevent subsequent perforation. Many studies have indicated that doxycycline may accelerate the healing of corneal wounds and inhibit the neovascularization and inflammation of the cornea after alkali burn [26, 33]. Our previous studies have investigated the effects of doxycycline on corneal neovascularization and inflammation after alkali burn [34, 35]. However, the molecular mechanism of doxycycline in corneal healing is unknown. In our current experiment, we explored the effect of doxycycline on TGF-*β*1, MMP-9, *α*-SMA, and NF-*κ*B expression to clarify its mechanism of action in corneal scarring.

Corneal wound healing is a complicated process involving molecular cascade events. The key to this healing is re-epithelialization, which involves keratinocyte proliferation, migration, differentiation, and extracellular matrix remodeling [36]. There are three dominant cell types in the cornea: surface epithelial cells, stromal keratocytes, and endothelial cells. These cells have common mechanisms to promote wound healing, including cell migration and proliferation, the involvement of growth factors and cytokines, and extracellular matrix (ECM) remodeling [36, 37]. In this study, the cornea presented with epithelial defects, edema, and unclear iris texture on day 3 after corneal alkali burn. The corneal opacity was lower in the doxycycline group than in the control group 7 and 14 days after chemical injury, and the cornea was almost transparent in the doxycycline group on the 14th day (Figure 1(a)). Doxycycline treatment also improved corneal wound healing (Figure 2(a)).

TGF-*β*1 is abundantly present in human tears, which are secreted by the lacrimal glands and are produced by epithelial cells and inflammatory cells located on the surface of the eye [38, 39]. When the cornea is injured, due to the absence of a basement membrane, TGF-*β*1 penetrates into the underlying stroma and initiates the differentiation of keratocyte subsets into myofibroblasts [40]. This process is key to the contraction and closure of corneal wounds [41]. However, the overexpression of TGF-*β*1 aggravates corneal damage, resulting in the accumulation of myofibroblasts in the stromal layer. This is believed to be the main cause of corneal haze postinjury [42]. The results of RT-PCR, ELISA, and Western blotting for TGF-*β*1 indicated that the expression of TGF-*β*1 in the cornea was noticeable after chemical injury. Compared with that in the control group, TGF-*β*1 expression was decreased in the doxycycline group on days 3, 7, and 14 after injury. Meanwhile, the results of immunofluorescent staining and Western blotting for *α*-SMA expression showed the same trend as that of TGF-*β*1 expression in the doxycycline group and the control group, which indicated that doxycycline inhibited the expression of TGF-*β*1 and *α*-SMA. In other words, doxycycline suppressed the expression of TGF-*β*1 and the formation of corneal scars.

MMP-9 plays a role in many ocular physiological processes, including angiogenesis and wound healing [43], and it has been found to be expressed only in rats with corneal injury but not in normal corneal stroma [44]. The presence of MMP-9 has been identified in the tears of patients with corneal alkali burns [45]. Furthermore, MMP-9 has been shown to promote the migration of basal epithelial cells, and its expression pattern is consistent with the remodeling of the subepithelial basement membrane region [46]. Kim et al. [47] treated primary human corneal epithelial cells stimulated by TGF-*β*1 with doxycycline, and the results showed that doxycycline inhibited the level of MMP-9 expression. In the present study, MMP-9 expression was also decreased in the doxycycline group, and thus, the subepithelial basement membrane may have been more intact in the doxycycline group than in the control group. On the other hand, the delayed corneal wound healing in the control group may have been due to the high expression of MMP-9.

NF-*κ*B is a major transcription factor that regulates many biological processes and is mainly involved in corneal wound healing, inflammation, angiogenesis, and the stress response [48]. Wilson et al. [49] found that the NF-*κ*B ligand is increased in the epithelium after corneal scraping and is also expressed in cultured corneal fibroblasts. Taheri and Bazan [16] showed that increasing the binding of NF-*κ*B to MMP-9 promotes upregulation of the MMP-9 expression in human corneal epithelial cells. However, Shin et al [20] reported that doxycycline inhibited TGF-*β*1-induced myofibroblasts via NF-*κ*B signaling pathways. In our study, the Western blot results showed that the expression of NF-*κ*B was lower in the doxycycline group than in the control group, which was similar to the pattern of expression of MMP-9 and TGF-*β*1. Therefore, we suspect that doxycycline may inhibit the expression of MMP-9 and TGF-*β*1 during corneal wound healing through NF-*κ*B signaling pathways.

In conclusion, treatment with doxycycline eye drops may accelerate wound healing in corneal tissue after alkali burn, and it may decrease corneal stromal haze by inhibiting MMP-9 and TGF-*β*1 expression. Doxycycline may reduce TGF-*β*1-induced myofibroblast transdifferentiation through the NF-*κ*B signaling pathway.

## Data Availability

The data used to support the findings of this study are included within the article.
